# Influences of photic stress on postsubicular head‐directional processing

**DOI:** 10.1111/ejn.13887

**Published:** 2018-03-25

**Authors:** Johannes Passecker, Md. Nurul Islam, Vincent Hok, Shane M. O'Mara

**Affiliations:** ^1^ Trinity College Institute of Neuroscience Trinity College Dublin Dublin 2 Ireland; ^2^ Aix‐Marseille Université CNRS UMR 7291 Marseilles France; ^3^ Present address: Center for Brain Research Medical University Vienna Spitalgasse 4 1090 Vienna Austria

**Keywords:** head‐direction cells, postsubiculum, presubiculum, rats, spatial navigation

## Abstract

The stress response serves vital adaptive functions. However, acute stress episodes often negatively impact cognitive processing. Here, we aimed to elucidate whether stress detrimentally affects the head‐direction cells of the postsubiculum, which may in turn impair downstream spatial information processing. We recorded neurons in the rats’ postsubiculum during a pellet‐chasing task during baseline non‐stress conditions and after a 30‐min acute photic stress exposure. Based on their baseline firing rate, we identified a subpopulation of head‐direction cells that drastically decreased its firing rate as a response to stress while preserving their head directionality. The remaining population of head‐direction cells as well as other neurons recorded in the postsubiculum were unaffected. The observed altered activity in the subpopulation might be the basis for spatial processing deficits observed following acute stress episodes.

## Introduction

The hippocampal formation (HF) of the mammalian brain (comprising CA1, CA3, dentate gyrus and the subicular region) has received a great deal of attention, as it is thought to be a central brain structure supporting memory processing and spatial navigation (O'Keefe, 1976; Eichenbaum, 2004; Aggleton *et al*., 2010). The subicular areas have not received as much attention, although they are an important output (Witter *et al*., 1990; O'Mara *et al*., 2001) as well as input structures (via the entorhinal cortex) (Kohler, 1985) of the HF.

The discovery of head‐direction (HD) cells in the postsubiculum (the dorsal part of the presubiculum) (Ranck, 1984) has sparked interest in its role in spatial navigation and spatial memory. The postsubiculum's strong afferent connectivity with the anterior thalamic regions and the medial septal nuclei (van Groen & Wyss, 1990; Jankowski *et al*., 2013), places itself within the spatial navigation circuit. HD cells are dependent on the orientation of the head in space, and their firing rates generally follow a Gaussian‐shaped tuning curve, with a single peak defining the preferred firing direction. Head direction in postsubicular neurons is independent of whether the head turns in a clockwise or anticlockwise manner towards the preferred firing direction (Taube *et al*., 1990a). Those cells are believed to provide a neuronal compass supporting navigation (Butler *et al.,* 2017). Since then, HD cells have been found in a wide variety of brain structures linked to the Papez circuit (Taube, 1998). The HD signal is believed to originate in vestibular inputs that are necessary for the head directionality of neurons in the anterior dorsal nucleus (Taube, 2007; Valerio & Taube, 2016). Anterior dorsal HD cells provide important inputs to the postsubiculum, where head‐directional information may be integrated with environmental information and, therefore, contribute to spatial memory processing in downstream structures (McNaughton *et al*., 2006). The firing of HD cells is thought to be an important component of the more complex spatial tuning of grid cells and place cells possibly via boundary vector cells (Knierim *et al*., 1995; Lever *et al*., 2009). HD cells discharge in respect to the rat's directional heading (Ranck, 1984; Taube *et al*., 1990b), whereas grid cells have multiple spatially selective firing fields, which form a well‐defined lattice believed to provide the basis of a metric used for calculating position (Hafting *et al*., 2005). Both head‐directional and grid systems are key components of the path integration process on which egocentric navigation is believed to be based (McNaughton *et al*., 2006).

The ventral parts of the subiculum have been shown to be involved in stress regulation (Herman & Mueller, 2006). Ventral subicular glutamatergic efferents innervate GABAergic neurons which in turn project to the paraventricular hypothalamic nucleus (Cullinan *et al*., 1993; Herman *et al*., 1995; Radley & Sawchenko, 2011). Via this route, ventral subicular neurons could provide modulatory input onto neurons influencing corticotropin‐releasing hormone and other HPA‐activating neuropeptides levels (Jacobson & Sapolsky, 1991; Cullinan *et al*., 2008). On the other hand, there is considerable evidence that stress might also have a direct influence on the spatial processing capabilities. In rodent models, a substantial amount of literature showed a range of spatial deficits with regard to uncontrollable acute stress (for review see Cazakoff *et al*., 2010; Kim *et al*., 2015; McEwen *et al*., 2016). These deficits are also observed in human subjects, who, under the influence of acute stress, experience a distortion of time and space that has been linked with egocentric cue processing deficits (Hancock & Weaver, 2005). We and others have provided evidence that altered firing properties of hippocampal place cells after stress exposure could contribute to the spatial memory deficits observed in freely behaving rats (Kim *et al*., 2007; Passecker *et al*., 2011). Additionally, there is evidence that the head‐directional signal is important for allocentric‐based navigation (Gibson *et al*., 2013) and for stabilizing hippocampal place cell activity (Calton *et al*., 2003). Given those results, we asked whether acute stress can affect spatial processing upstream of the hippocampus and studied the effects of uncontrollable acute stress on the head‐directional system of the postsubiculum.

We identify here a subpopulation of head‐direction cells with substantially decreased firing rates in response to stress, while preserving their head directionality. The remaining population of HD cells as well as other neurons recorded in the postsubiculum were unaffected. This subpopulation‐dependent altered activity might therefore constitute a basis for spatial processing deficits observed during acute stress episodes.

## Experimental procedures

### Ethics

Six (4–6 months) male Wistar rats (B&K, UK) weighing between 420 and 530 g were used. Upon arrival, animals were housed individually and handled by the experimenter daily for a week before being trained in the pellet‐chasing task (see below). Rats were food‐deprived to 90% of their *ad libitum* body weight, kept in a temperature‐controlled laminar airflow unit and maintained on a 12‐h light/dark cycle (lights on from 08:00–20:00 hours). Experiments were carried out in strict accordance with the European Union directives on animal experimentation (2010/63EU), and the Cruelty to Animals Act, 1876, and followed international guidelines of good practice. All experiments were approved by the responsible Ethical Committee, the Bioresources Ethics Committee, Trinity College, Dublin, Ireland. As the photic stressor was stressful, we aimed to reduce the number of exposures and number of animals while maintaining meaningful results (recorded HD cells) to allow valuable statistical analysis. The prediction of neurons to be recorded per animal cannot be reliably estimated prior to surgeries, we recorded and implanted rats sequentially to allow for a better estimation. We refined our recording procedure by increasing the tetrode bundles from four to eight during the experiment to allow for a higher yield per animal and thus reduce *n* numbers.

### Surgical procedures

Surgical protocol and recording techniques followed the detailed descriptions in Brotons‐Mas *et al*. (2010) and Tsanov *et al*. (2011). Anaesthesia was induced using intraperitoneal injections of ketamine (100 mg/kg) and xylazine (10 mg/kg). Anaesthesia was kept with a constant flow of isofluorane (2%) and maintained for the complete duration of the surgery. Enrofloxacin (10 mg/kg, s.c.), methylprednisolone (10 mg/kg, i.p.) and buprenorphine (0.05 mg/kg, s.c.) were administered to reduce the risk of infection and inflammation as well as for analgesia, respectively. Rats were mounted in a stereotaxic apparatus (Kopf Instruments, CA, USA) via ear bars and the skull exposed. Six to seven jewellers’ stainless steel screws were fixed into the skull to provide later support for the microdrives. One of the screws acted as grounding point. Craniotomies and duratomies were performed, and rats were implanted with bundles of four or eight tetrodes of platinum–iridium wires (California Fine Wire Ltd., USA) mounted onto small driveable microdrives (Axona Ltd., UK). The following coordinates were used 6.2 mm posterior to bregma (up to −6.7 mm), ~2.7 mm lateral to the midline and at least 2 mm dorsoventral from the dura (Paxinos & Watson, 1998). Once implanted, the craniotomy was covered with Vaseline and the microdrive base cemented into place with dental acrylic (Associated Dental, Swindon, UK). To avoid further infections of surrounding tissue, topical antibiotic powder (Cicatrin^®^, Wellcome, Ireland) was dusted onto the tissue and animals were allowed at least a week of recovery. During the first 3 days, animals were maintained under an antibiotic and analegesic drug regime to ensure full recovers. Once animals recovered, animals got habituated to the room, experimenter and the handling procedure (twice daily for three days as a minimum).

### Extracellular electrophysiology

Once animals were habituated, screening procedure started and tetrodes were slowly advanced until recordable units were found in the respective area (maximal rates 25–50 μm/day). Tracks were verified post‐mortem. Sixteen‐minute long recordings were performed in a square‐shaped arena (64 × 64 × 25 cm) located in the centre of a room with one distal visual cue fixed on the surrounding curtain. 20 mg food pellets (TestDiet™ 5TUL formula) were delivered into the arena at random locations ca. every 20s by the experimenter. Single‐unit identification was based on several criteria. First, neurons had to be active in any two consecutive recordings (control and control, or control and stress) and had to present same waveform characteristics (amplitude, height and duration, assessed by a Wilcoxon rank‐sum test) in both sessions. Additionally, they had to present at least a minimum firing frequency of 1 Hz in one of the two sessions. Furthermore, units had to demonstrate a clean refractory period (<2 ms) in the autocorrelogram. Rats had to explore at least 90% of all bins in their first control session before another control or stress session was performed. Once a complete set of recordings (control + control, or control + stress) have been performed, tetrodes were advanced a maximum of 40 μm. Daily record of tetrode advancement was kept for verification purposes. At the end of the experiments, rats were euthanized by guillotine and their brains collected for electrode position verification. Brains were collected and stored in a 4% PFA solution (Sigma, UK), embedded in paraffin, and the target region was microtome sliced into 20 μm sections. Thereafter, brain sections were stained with a standard haematoxylin and eosin staining protocol to allow cellular identification. Tetrode tracks were verified by visual inspection of the experimenter under a standard microscope (Leica, GER) fitted with a 5× and a 20× lens (Leica, GER).

### Photic stress procedure

The inescapable acute stress protocol consisted of 30‐min photic exposure. Rats were placed into a black round bucket while being exposed to bright light with approx. 120 cd at the bucket floor. The light source was approx. 60 cm in distance resulting in an exposure of approx. 330 lux. Although this procedure is considered a mild stressor, this stress protocol has consistently induced stress responses and spatial memory deficits (Commins & O'Mara, 2000; Passecker *et al*., 2011) as well as performance deficits in spatial memory in Wistar rats (Passecker *et al*., 2014). Thus, it has been used in our laboratory as well as in other laboratories as a reliable inducer of a systemic behavioural stress response (Katz *et al*., 1981; Toledo‐Rodriguez & Sandi, 2007; Nathiya & Vanisree, 2010). Following the bright light exposure, rats were then allowed 30 min of rest in the home cage, before a second (16 min) recording in the pellet‐chasing task commenced in the same environment as the original control recording. The delay period of 30 min following the bright light served two main purposes. First, to reduce possible short‐term visual impairments and secondly, a reliable activation of glucocorticoid and mineralocorticoid pathways as described in literature (Oitzl *et al*., 2010). Animals were never stressed twice within a three‐day period to decrease the possibility of adaptation to the stressor and to prevent chronic stress induction. On average, each animal was exposed to the photic stressor a total of approx. 8.2 times usually over several weeks. For control experiments, we recorded consequently the animal in the same environment with a similar timeline as during stress experiments. The second control session started a minimum 1 h after the first control session finished. Animals were kept in the home cage (in a separate room) within that period and not in the bucket without light. We wanted to exclude context specific or odour specific stress reactions associated with the stress bucket. Hence, during control experiments, the timeline was as follows: 16‐min control1 recording followed by 1‐h rest in the home cage followed by another 16‐min long control2 recording (same environment as control1). The timeline for the stress recording, on the other hand, was as follows: 16‐min control1 recording followed by 30 min. of bright light exposure in a bucket, followed by another 30 min. of no bright light in the home cage ending with a 16‐min recording in the same environment as control1.

### Statistical analysis

Head direction was calculated from the relative positions of two LEDs. The total time and the number of spikes discharged at each head direction during a recording session were summed from the collected samples. Head‐direction cell firing rate was determined by dividing the number of spikes in each angular bin by the time spent in each angular bin. The minimum angle based on the chosen resolution of 60 angular bins was 6 degrees. The mean head‐directional firing rate was defined as the circular mean of the firing rate in Hz. The head‐directional half‐width was calculated as the width of the tuning curve measured at 50% of the peak firing rate in degrees. Maximum peak firing rate was defined as the firing rate in Hz within the angular bin at the peak of the directional tuning curve. The angular bin with the highest firing rate was defined as the peak head direction. Another measure was the mean (preferred) head direction (in degrees) of the cell that was based on the circular mean of the degrees of the directional tuning. The theta index correlation describes the Pearson correlation goodness of fit between the fitted autocorrelation (Royer *et al*., 2010) and the autocorrelation itself. A low value describes a minimal fit and thus low or bad theta frequency modulation of the cells. Speed correlations were based on 200‐ms time bins which were correlated with the firing rate in the respective bins.

For the classification of the neuronal recordings, we performed a multiple regression analysis (MRA) with speed, angular velocity, head direction, spatial information as well as border activity (defined as variance in firing rate according to the distance from the border area) as variables. Calculated partial *r* ^2^ values explaining the variance of the firing rate were used to define possible subpopulations and compared against each other using a cumulative distribution analysis. Partial correlational values were obtained for each individual cell and each variable, respectively. The supervised k‐means clustering was tested on a range of possible cluster numbers (1 to 10) and its performance evaluated by the silhouette criterion (Rouseeuw, 1987). This approach allowed us a non‐biased way to estimate the optimal cluster number.

We analysed and visualized session‐to‐session data as scatter plots with linear regressions. This form of data representation allowed us to show all variable changes between sessions and the raw data in full. Additionally, we systematically performed non‐parametric Wilcoxon‐signed rank tests to assess statistical significance (alpha set at 0.05) between conditions as all sample distributions were not normally distributed (Kolmogorov–Smirnov test). All statistics were performed using Matlab R2014a^®^ or SigmaPlot13.0^®^. Neuronal unit activity was analysed offline and clustered offline with the TINT software suite (Axona Ltd., UK). Data processing was performed by custom‐written Matlab code using the NeuroChaT^®^ software package.

## Results

**Figure 1:**
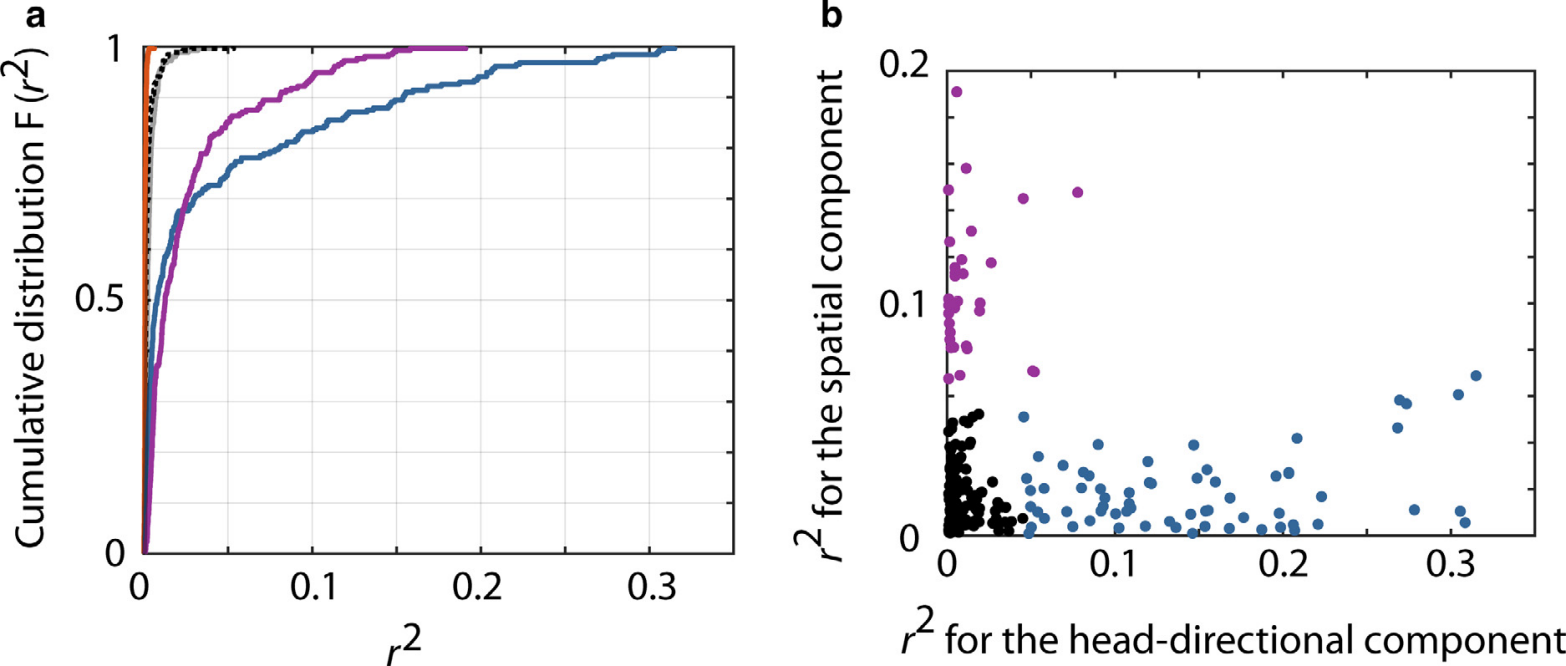
Identification of head‐direction cells and characterisation of recorded postsubicular cells. (a) Multiple linear regression analysis revealed that only spatial (purple) and head‐directional information (blue) provides significant influence over postsubicular firing rates. Border (black dashed), speed (grey, behind the black line) as angular velocity (orange) information failed to significantly influence the firing rate (b) a supervised clustering revealed three clusters. One cluster with strong spatial information correlation (purple), one with a significant head‐directional component (blue) and one cluster which neither correlated with spatial nor head‐directional information (black). [Colour figure can be viewed at http://www.wileyonlinelibrary.com].

In total, 230 well‐defined neurons were recorded from the dorsal postsubiculum which could be successfully matched across two consecutive conditions. To identify head‐directional coding in an unbiased fashion while accounting for border, angular velocity, speed and spatial variables, we performed a multiple regression analysis on each cell to test for head‐directional correlations. We did not find significant correlations for angular velocity, speed or border information correlations (Fig. 1a). Spatial and head‐directional correlations were most prominent (Fig. 1a). To separate or identify cells that carry both prominent head‐directional and spatial information, we performed a supervised k‐means clustering on all cells using their partial correlation values for the spatial and head‐directional variable. The analysis revealed three clearly distinct clusters (Fig. 1b). Sixty‐five head‐direction cells were identified by the *k*‐means clustering which were used for in‐depth analysis.

**Figure 2:**
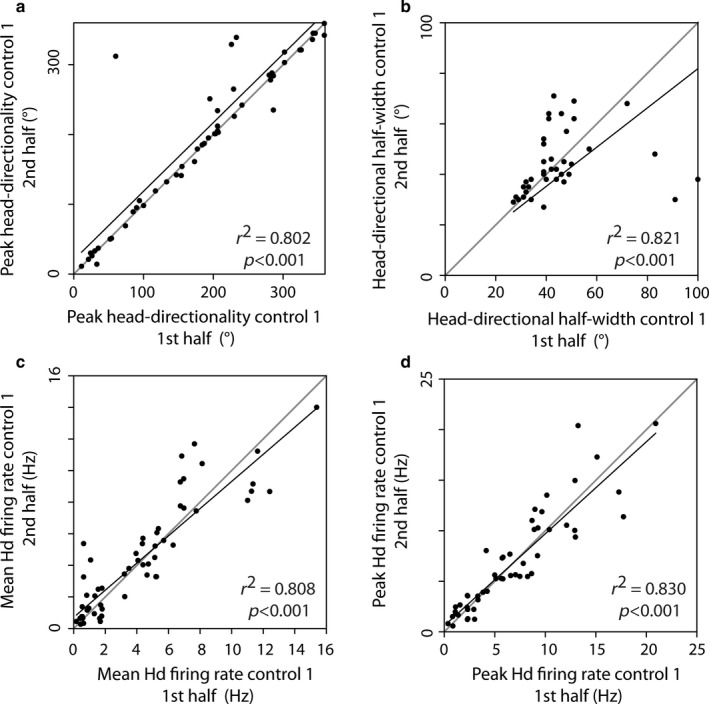
Within‐session reliability is conserved for head‐direction cells of the postsubiculum. Both head‐directional tuning (a) and its curve (b) are highly correlated between the first and second half of a control recording. The same holds true for the mean head‐directional firing (c) rate as well as the peak head‐directional firing rate (d). Black lines represent regression lines. Grey lines depict ideal correlation. Each panel can be divided into two regions. Any data localised in the region above the grey line (top left triangle in subsequent legends) would show an increase in the variable in the condition depicted on the *y*‐axis (in this case, the second half of the session). Any data localised in the region below the grey line (bottom left triangle in subsequent legends) would show a higher value of the variable in the condition depicted on the x‐axis (first half of the session).

**Figure 3:**
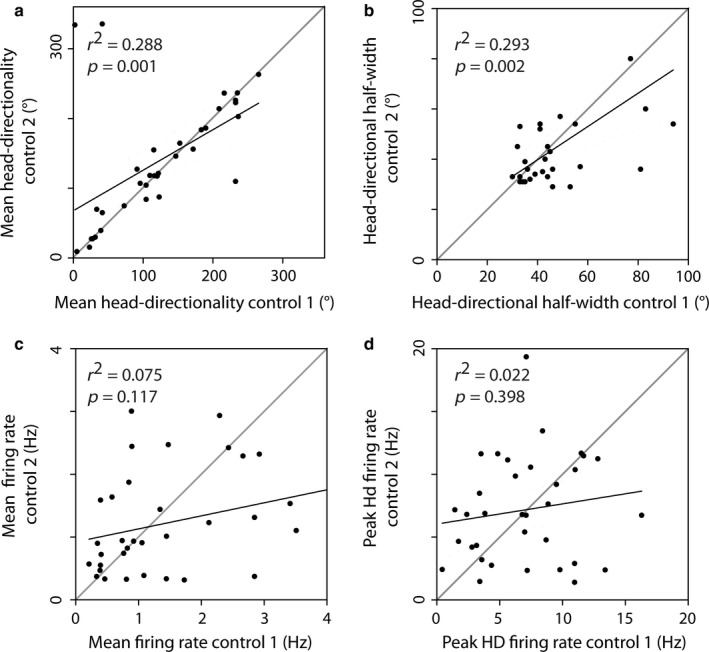
Reliability of head‐direction cells across consecutive control sessions. Head‐directional tuning (a) as well as the accuracy of the head‐directional tuning curve (b) is preserved in cells between control sessions. Mean firing rate of the cells (c) as well as peak head‐directional firing rate (d) is not significantly correlated between sessions on the population level. Black lines depict regression lines. Grey lines depict ideal correlation. Top left triangle presents increases in variable towards the second control session, whereas the bottom left triangle presents higher variable values in control session 1.

To assess the stability of those cells, we analysed the within‐session variance of the main head‐directional variables between the first half of the control session and the second half of the same control session. As expected, all major head‐directional variables remained highly significantly correlated within a freely behaving recording session (Fig. 2). Stable head‐directional variables within a session did not necessarily prerequisite stable representations of the same cells across sessions. We therefore assessed whether the head‐directional variables remain constant between two consecutive control sessions. Figure 3 depicts that both mean head directionality (Fig. 3a) and the head‐directional half‐width (Fig. 3b) remained significantly correlated and not significantly different (mean head directionality: *Z*_34_ = −0.795, *P *=* *0.432; mean head directional half‐width: *Z*_30_ = 1.178, *P *=* *0.244). The peak head directionality (*Z*_34_ = −0.569; *P *=* *0.576) as well as the head‐directional partial *r* values (*Z*_34_ = −0.849, *P *=* *0.401) did not significantly differ between both control sessions. Although there was no overall effect on the mean head‐directional firing rate (*Z*_34_ = −0.862, *P *=* *0.394) and the peak firing rate (*Z*_34_ = −0.331, *P *=* *0.748), both firing rates increased their variance and lost their significant correlation between two control sessions (Fig. 3c, d).

**Figure 4:**
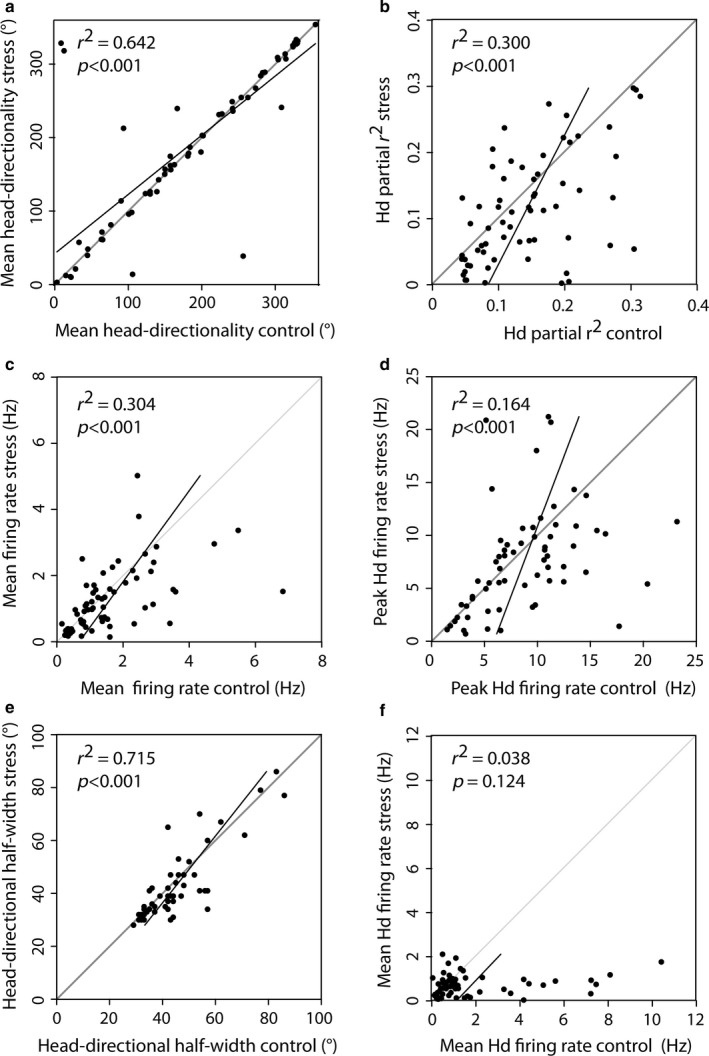
Loss of head‐directional information of subicular head‐direction cells after stress exposure. After a stress exposure, a significant correlation with prior control values could be observed for the mean head‐directionality, head directional correlation information, overall mean firing rate, peak head‐directional firing rate and head‐directional half‐width (a–e). Only the mean head‐directional firing rate (mean of the directional tuning curve) (f) did not significantly correlate with the control values. Black lines represent regression lines. Grey lines depict ideal correlation. Top left triangle presents increases in variable towards the control session, whereas the bottom left triangle presents higher variable values in the stress session.

Following the assessment of head‐direction cells in two consecutive control sessions, we studied the effects of photic stress on the 65 identified head‐direction cells. For that purpose, we first performed a control recording session and exposed the animals 30 min to photic stress. The animals were then allowed a 30‐min resting phase and then recorded a second time in the same environment as during the first control recording. The preferred head directionality of units remained unaffected (Fig 4a, *Z*_65_ = −0.224, *P *=* *0.826) between the two conditions, indicating each unit remained a stable predictor of direction. Head‐directional partial *r* values (Fig. 4b), mean firing rate (Fig. 4c), peak head‐directional firing rate (Fig. 4d), mean head‐directional half‐width correlations (Fig. 4e) between control and stress conditions remained significant, but in all cases, the variables showed a significant decrease in value (mean head‐directional partial *r* value *Z*_65_ = −3.029, *P *=* *0.002, mean head‐directional half‐width *Z*_56_ = −2.493, *P *=* *0.013, peak head‐directional rate *Z*_65_ = −2.109, *P *=* *0.035, mean firing rate *Z*_65_ = −2.153, *P *=* *0.032). Especially, apparent is the highly significant decrease for the mean head‐directional firing rate (*Z*_65_ = −4.053, *P *=* *5.1e‐05) and its loss in correlation between sessions (Fig. 4f).

**Figure 5:**
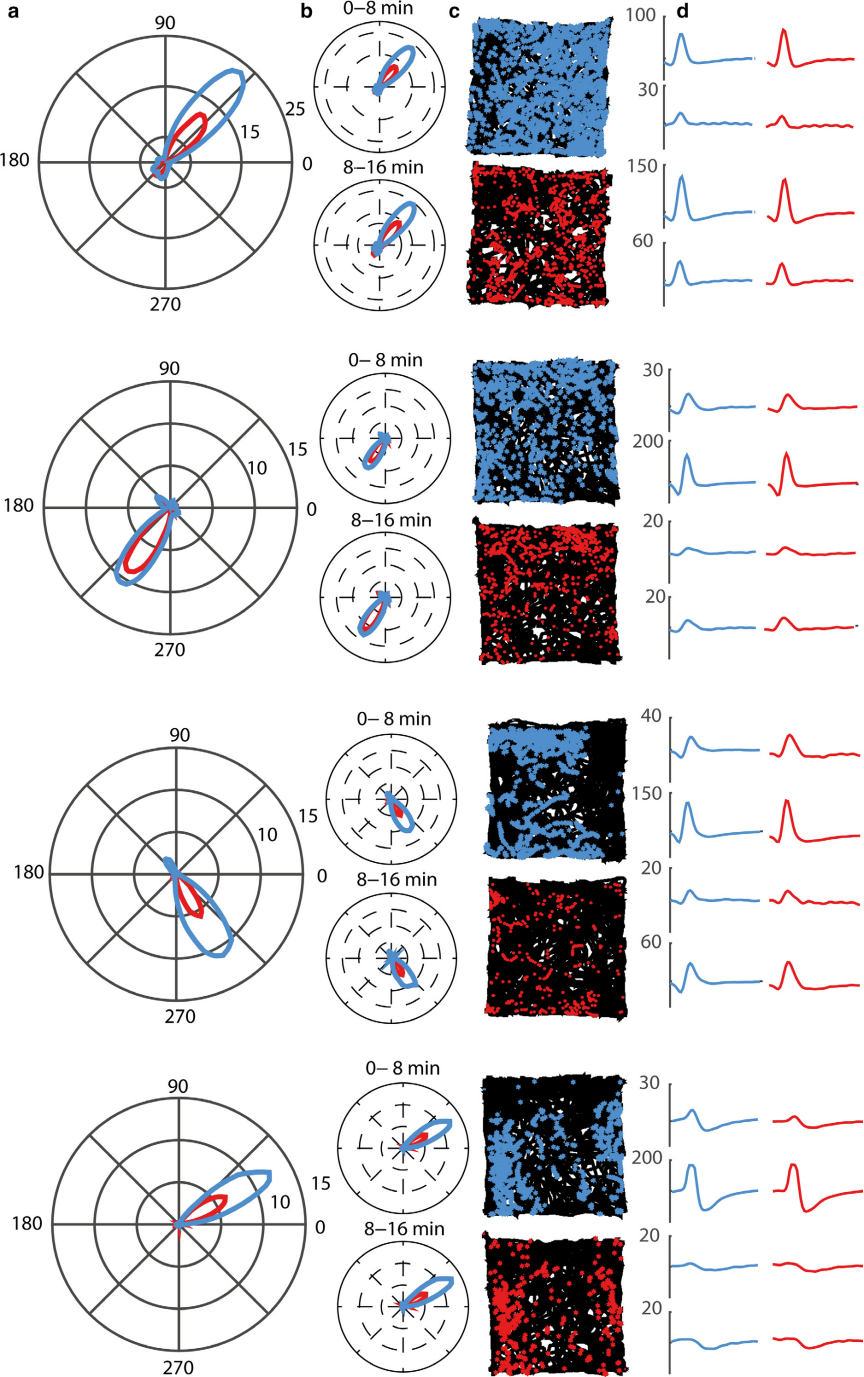
Examples of firing rate changes in head‐direction cells after stress. Four example neurons with clearly defined head‐directional firing rate dependencies. (a) 360° plot depicting the main preferred head‐directional firing of the cell. Blue denotes the control condition, red the stress condition. All examples represent the population with a marked decrease in the respective firing rate. (b) Preferred directional heading of the neurons remains constant during the recording for both conditions. Additionally, the decrease in firing rate appears to be a global phenomenon throughout the recording time of 16 min. (c) Spiking activity (respective color) and path (black) for both conditions and each neuronal example. (d) Mean spike waveforms for both conditions indicate that there has been no tetrode bundle drift between the recording sessions (*y*‐axis in μV; *x*‐axis shows 50 sample points). [Colour figure can be viewed at http://www.wileyonlinelibrary.com].

Individual cell examples can be observed in Fig. 5a–d, representing a distinctively preserved directionality, while the firing rate showed marked decrease after stress exposure. To identify whether the firing rate changes were specific for the head‐direction neuronal population or a general effect across the postsubiculum, we tested the firing rates between the control and stress conditions for the remaining population and did not find any significant difference (*Z*_95_ = −0.186, *P *=* *0.854; *r* ^2^ = 0.571, *P *<* *0.001). This indicated that firing rate changes observed for head‐direction cells were not similarly present in the remaining population ruling out the possibility of a non‐specific global inhibition effect of the postsubiculum.

**Figure 6:**
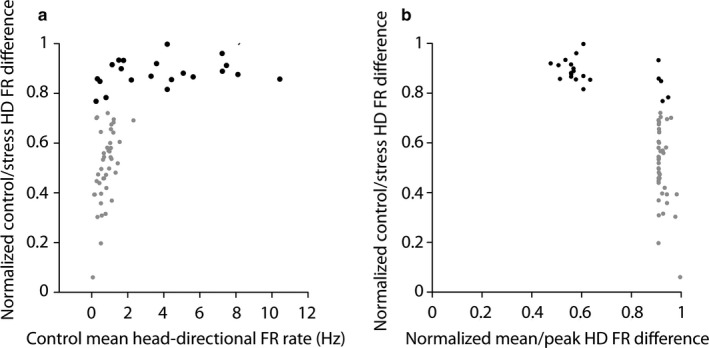
Subpopulation of HD cells decrease its firing rate as a response to stress. (a) Cells that decreased their mean head‐directional firing rate more than 75% after stress were selected (black dots) and compared to all other head‐direction cells (grey cells) and plotted vs. the control mean head‐directional firing rate. (b) The same cells were plotted against the mean HD firing rate divided by the peak head‐directional firing rate during the control session.

To address whether the firing rate changes in the stress condition were correlated with changes in behaviour, we assessed speed and angular velocity changes between both conditions. Angular velocity was not significantly changed between both conditions (*Z*_65_ = −0.167, *P *=* *0.870). Speed did significantly differ between the control and stress conditions (*Z*_65_ = −3.737, *P *=* *1.8e‐4). However, speed changes may not be related to the observed firing rate‐related changes. When we restricted the analysis to sessions where speed did not significantly differ between control and stress conditions (*Z*_46_ = 0.432, *P *=* *0.670; median control: 17.236 vs. median stress: 17.341), the firing rate for the respective cells in those sessions remained highly significantly decreased (*Z*_46_ = −3.392, *P *=* *6.9e‐4). As our analysis of behavioural variables did not result in a plausible explanation, we attempted to assess whether the photic stress influences a specific subpopulation of head‐direction cells. We found a good predictor of future firing rate decrease in head‐direction cell based on the firing activity of these cells during the control session (Fig. 6). Indeed, a major change in mean head‐directional firing rate (black dots) due to the stressor was predicted by a higher *a priori* control firing rate (Fig. 6a). Those cells with the highest firing rates variations presented a relatively low ratio between their mean head‐directional firing rate and their peak firing rate compared to the remaining head‐directional population, which have a higher ratio of peak to mean head‐directional firing rate (Fig. 6b). Interestingly, the head‐direction cells that were affected significantly by the stressor were only weakly correlated with the theta rhythm, when compared to the remaining population of head‐direction cells (*U*_21/42_ = 237, *P *=* *0.003). Here, we found a decreased Pearson correlation describing the decreased goodness of fit for the sinusoidal equation to the auto‐correlational histogram for the specific subpopulation of HD cells. We then went back to those cells and assessed whether their spike shape characteristics differ as well which would provide more insight if the stress‐affected cells could indeed describe a more coherent subpopulation. Indeed, both characteristics of spike shape differed significantly. First, mean spike width differed significantly (*U*_21/41_ = 264; *P *=* *0.010) with a broader shape for stress‐affected cells. Additionally, spike amplitude decreased for the same population significantly (*U*_21/41_ = 296; *P *=* *0.035).

We also performed a regression analysis to test whether the session number (reflecting the number of exposures to the stressor) could explain the degree of the firing rate change in the head‐directional cell population. Our results show that there is a weak but significant correlation between those two factors with an *r* ^2^ value of 0.089 and a *P*‐value of 0.017. This last result indicates that with increasing exposure, the likelihood of a firing rate decrease diminishes, suggesting, therefore, a stress adjustment by the animal, which is to be expected.

## Discussion

Stress can markedly affect brain function in both a positive and negative fashion, as well as in a cognitive‐domain‐dependent manner. Here, we show that short episodes of photic stress can cause deficits in head‐directional cells in the postsubiculum: a distinct subpopulation of head‐directional cells showed marked decreases in head‐directional firing rate. This change in the firing regime may affect information processing through a loss of correlation of the directional component of neuronal activity.

Between the two control conditions, head‐direction cells maintained their head directionality. However, they presented a higher variability in firing rate‐related variables than expected. Importantly, the firing rate variation between control sessions had no specific direction; that is head‐direction cells could either decrease or increase their firing activity during the second control session. Contrasting with this, stress resulted in a significant shift towards a decrease in head‐directional firing rate and towards a decrease in the head‐directional correlation content. These results indicate therefore a loss of informational value of the neuronal firing rate after stress exposure. Those changes could not be ascribed to changes in behaviour (speed or angular velocity) and were specific to head‐direction cells only. Additionally, only a subset of higher firing rate and lower theta correlated cells were detrimentally affected. The remaining head‐direction population did not differ from the variability in firing rate one would expect from the control recordings.

Due to the nature of our tetrode recordings, we cannot deduce with certainty the layer specificity of our recorded head‐direction cells. However, results from a recent study (Preston‐Ferrer *et al*., 2016) showing a decreased theta rhythmicity specific to layer 3 postsubicular neurons (compared to layer 2 neurons) are consistent with our own data. Layer 3 neurons were found to predominately project to the medial entorhinal cortex, whereas layer 2 neurons targeted the retrosplenial cortex, as well as contralateral presubiculum. Although we cannot draw strong conclusions from this comparison, those layer 3 cells would fit well with our observations and link them with medial enthorhinal cortex‐projecting cells.

How the observed firing rate changes can affect spatial navigation or information flow of spatial information remains to be elucidated. An earlier study by Dudchenko & Taube (1997) has correlated head‐directional firing shifts with correct behavioural choices by shifting distal cues in accord with reward positions on a radial arm maze. In correct trials when the reward location was shifted about 90°, head‐direction cells shifted accordingly. Later, seemingly, contradictory studies (e.g. Golob *et al*., 2001) have led to the conclusion that animals have to associate the allothetic cue with the reward, preventing the rats’ response in accordance with the head‐directional shift of the head‐direction cells. Additionally, a more recent study has shown that postsubicular lesions can induce navigational deficits in rats (Peckford *et al*., 2014) under certain conditions and that the postsubiculum is necessary for spatial alternation on a t‐maze (Bett *et al*., 2012). Taken together, these data provide clear support to the idea that impaired or less‐specific head‐direction signals may cause navigational deficits. However, as we focused our study on neuronal firing properties, it is still unclear if they alone can lead to behavioural and navigational deficits. In our data, the loss of correlation between head‐direction signalling and neuronal firing rate indicates only an information loss which may be transmitted to downstream targets. The findings are in concert with the idea that stress can enhance cognitive processing of stress‐ or threat‐related information and help in its consolidation but at the expense of other cognitive systems possibly for a prolonged period (Quaedflieg & Schwabe, 2017). In the hippocampus, reports have shown that LTP can be initially enhanced followed by a prolong long‐lasting suppression effect (Akirav & Richter‐Levin, 1999; Wiegert *et al*., 2006) reinforcing first threat or stress‐related information followed by possible information processing deficits. However, it remains to be seen if similar strongly time‐dependent results apply also to the subicular areas in terms of LTP, synaptic strengthening and overall excitability. For example, the same stressor that impairs hippocampal memory enhances activity of non‐hippocampal areas important for habit or fear‐related behaviours in the caudate nucleus or amygdala (Kim *et al*., 2001; Schwabe & Wolf, 2012).

We report here for the first time that even in stable environments, postsubicular head‐direction cells show an overall degradation of head‐directional information processing, while sparing preferred directional tuning after acute stress exposure. These results cannot be explained by repeated exposures to the same environment alone or overt changes in behaviour. However, the causal relationship between these changes and putative navigational deficits remains to be tested. We cannot exclude the possibility that the cells most affected by stress are targets of visual processing streams from the visual cortex. Retinal damage or changes in rhodopsin levels have been only observed in much higher lux regimes and for significantly longer periods of continuous exposure in albino rats (e.g. Li *et al*., 2003; Tanito *et al*., 2008). Nevertheless, photic stress could potentially have lasting effects on the visual processing of environmental information translated to downstream target sites. Pérez‐Escobar *et al*. (2016) reported that a loss of visual input (i.e. during darkness session) resulted in a severe loss of head‐directional selectivity in the MEC and parasubiculum. Further studies will be needed to elucidate the role of this circuit under more challenging and diverse learning and stress conditions (e.g. Rahman *et al*., 2016) and will add valuable information to our understanding of stress effects on our cognitive processing capabilities.

## Conflict of interest

The authors declare no competing financial interests.

## Data & code availability

The data and code that support the findings of this study are available from the corresponding authors upon reasonable request.

## Author contributions

J.P. carried out the experiments and led the analysis of the paper in collaboration with M.N.I., and V.H. J.P. and S.M.O. planned the experiments and wrote the paper with editorial input from all authors. S.M.O. supervised all aspects of the work.
