# Supplementary material for: Influences of photic stress on postsubicular head‐directional processing
Source: Eur J Neurosci. 2018 Mar 25;47(8):1003–12. doi: 10.1111/ejn.13887 (PMC5947620; doi:10.1111/ejn.13887)
Supplement: Supplementary file 1 [file EJN-47-1003-s001.pdf]

EJN European Journal of Neuroscience FENS Federation of European Neuroscience Societies

Influences of photic stress on postsubicular head-directional processing

Johannes Passecker, Md Nurul Islam, Vincent Hok & Shane M. O'Mara

Review timeline:

Submission date:	13 November 2017
Editorial Decision:	18 December 2017
Revision received:	24 January 2018
Editorial Decision:	23 February 2018
Revision received:	25 February 2018
Accepted:	26 February 2018

Editor: Marco Capogna

1st Editorial Decision 18 December 2017

Dear Dr. Passecker,

Your manuscript has been reviewed by external reviewers as well as by the Section Editor, Dr. Marco Capogna, and ourselves. Based on these reviews, we regret to inform you that we are not able to accept your manuscript for publication in EJN in its present form. However, the research described in your manuscript is potentially of interest and therefore we invite you to resubmit a much revised version.

Specifically, one Reviewer and Section Editor raised serious concerns with the photic stress as model system. At present it is impossible to determine whether the observed effects are due to the stress response, or to the acute photodamage of retinal cells. We thus consider it necessary to provide data that would enable you to distinguish between systemic stress response and the acute retinal photodamage. Reviewer 1 raises issues of animal welfare. Please address each of their points carefully.

We also noted the following points that should be addressed in your revised version.
- Ethical approval for the experiments needs to be explicitly stated.
- Include details of the surgical anesthesia & peri-operative care.
- Methods section is quite hard to follow and would benefit from sub-headings.
- How were animals euthanised?
- Please proof-read reference list to ensure it adheres to to EJN style.

Thank you for submitting your work to EJN.

Kind regards,

Paul Bolam & John Foxe
co-Editors in Chief, EJN

Reviews:

Reviewer: 1 (Enrico Alleva, Istituto Superiore di Sanità, Italy)

Comments to the Author
I only comment two aspects: 1) the bioethical aspects, since rats are in stressing and painful situation, therefore a paragraph detailig the 3Rs principle applied to this experiment is necessary (why uses only 6 animals, eg?) 2) the stress procedure and the stress data discussion.
For the latters:
lines 26-27, acute photic exposure is a reconisedly standardised procedure?

63: stress regulation, how? Details are necessary, as well more quotations.
83-103: painful and stressing procedures, see above.
153: non normality of the data needs to be measured, not simply assumed.
215 p=0,854 ?
252-3: a paragraph on eustress needs to be added.

Reviewer: 2 (Rafal Czajkowski, Nencki Institute of Experimental Biology, Poland)

Comments to the Author
The submitted manuscript describes changes in the properties of head direction neurons in the rat postsubiculum upon acute photic stress. The authors provide fairly exhausting comparison of neuronal population consisting of 65 HD cells from 6 animals. The head direction tuning properties remain stable under these conditions, but the average firing rate drops in a subpopulation of recorded cells. The high baseline firing rate is a good predictor of the susceptibility to the stress effect. This is a very interesting finding and the quality of electrophysiological data analysis is more than satisfying.
There is one fundamental problem with the design of the experiment. The authors use photic stress as their model. Judging from the introduction and methods, the goal is to achieve a systemic stress response (lines 15-116). Unfortunately this type of stimulus influences not only the systemic pathways, but also one of the main inputs that drive the head direction neurons – the visual system. The Wistar rats are albinotic and their eyes are extremely sensitive to light stimulation. They do not possess melanin and even a relatively small light exposure causes irreversible damage to their retina. The albino rat has been a model system for retinal damage testing, starting from the classical paper by Noell (Noell 1966). A fairly recent review article neatly summarizes these studies (De Vera Mudry 2013). In the light of these findings it is impossible to determine whether the observed effect is due to the stress response, or to the acute photodamage of retinal cells. The authors provide only a two sentence comment to explain this possibility (lines 299-301). Also, they observe an adaptation effect upon consecutive sessions and explain it as adjustment to stress. An obvious alternative explanation is the decreased sensitivity of retina to the stimulus due to photodamage.
The electrophysiological data deserve publishing, but the design of the experiments does not provide satisfactory explanation of the observed effect. It is absolutely necessary to perform experiments that would distinguish between systemic stress response and the acute retina photodamage.
The very basic control would be the histological comparison between the retina of control animals (a parallel cohort never subjected to photic stress) and the experimental rats.
The authors could use different stressors (for example auditory) and compare their effects with the photic stress.
Another type of experiment would be to record HD activity in light vs dark conditions in order to identify visually driven HD cells, and then check whether this population is specifically affected by photic stress. If this was the case, the data would nicely support the concept that two populations of HD neurons may exist in parasubiculum, one responsible for the idiothetic input, and the other processing the allothetic stimuli provided by the visual system.

Authors' Response 24 January 2018

Detailed response to the reviewers in blue:

Reviewer: 1
Comments to the Author
I only comment two aspects: 1) the bioethical aspects, since rats are in stressing and painful situation, therefore a paragraph detailig the 3Rs principle applied to this experiment is necessary (why uses only 6 animals, eg?) 2) the stress procedure and the stress data discussion.

We thank the reviewer for pointing this out and we now provide more details of bioethical aspects which are of course a key element of the procedures. As outlined in more detail (see Reviewer 2 section), our photic light stress was mild with approx. 330 lux of exposure. This was based on previous experiments in-house where we had reliable defecation and urination, partly freezing behavior and erection of the hair and observed cognitive deficits (refinement). As the photic stressor was stressful and as such we aimed to reduce the number of exposures and number of animals while maintaining meaningful results (recorded neurons, and in particular recorded HD cells) to allow valuable statistical analysis. As the prediction of neurons to be recorded per animal cannot be reliably estimated, we recorded and implanted rats

sequentially to allow for a better estimation (reduction). Also we increased the tetrode bundles from 4 to 8 during the experiment to allow for a higher yield per animal (reduction & refinement). We consider the model system rat already as a replacement when one wants to study head-directional tuning in the postsubiculum. We have added a paraphrased version as paragraph to the methods section.

For the latters:
lines 26-27, acute photic exposure is a reconisedly standardised procedure?

We are uncertain to what specifically the reviewer refers so we try to answer two interpretations: (1) The acute photic stress protocol is not incorporated into the IACUC standard procedures, but we are not aware that any acute stress procedure applied in the literature is. (2) Bright light exposure as a mild stressor has a long history and has been widely applied beyond what we have referenced. e.g. Xu, Anwyl & Rowan, 1997, Nature; Commins & O'Mara, 2000, Psychobiology or slightly adapted in many other studies e.g. Kalueff & Tuohimaa, 2004, Weinstock et al. 1992.

63: stress regulation, how? Details are necessary, as well more quotations.

We have now expanded this section of the discussion and included more references to support that statement. See lines64 to 68 and respective references

83-103: painful and stressing procedures, see above.
153: non normality of the data needs to be measured, not simply assumed.

We apology for the inconsistency, by assuming non-normality based on our sample size we wanted to be conservative per se. In fact, all non-parametric tests were run in Sigmastat and were automatically tested first, for normality which they have failed. To be sure we have rerun all tests again and confirmed the non-normality of the data and have now included an additional sentence in the Methods section.
215 p=0,854 ?

We thank the reviewer for spotting the mistake and have corrected it.
252-3: a paragraph on eustress needs to be added.

An important point addressed by the reviewer, we have now added a section on the positive effects of stress including additional references.

We hope that the added sections and details are satisfying and we believe they are important and improve the manuscript and the understanding of thereof.

Reviewer: 2

Comments to the Author
The submitted manuscript describes changes in the properties of head direction neurons in the rat postsubiculum upon acute photic stress. The authors provide fairly exhausting comparison of neuronal population consisting of 65 HD cells from 6 animals. The head direction tuning properties remain stable under these conditions, but the average firing rate drops in a subpopulation of recorded cells. The high baseline firing rate is a good predictor of the susceptibility to the stress effect. This is a very interesting finding and the quality of electrophysiological data analysis is more than satisfying.
There is one fundamental problem with the design of the experiment. The authors use photic stress as their model. Judging from the introduction and methods, the goal is to achieve a systemic stress response (lines 15-116). Unfortunately this type of stimulus influences not only the systemic pathways, but also one of the main inputs that drive the head direction neurons – the visual system. The Wistar rats are albinotic and their eyes are extremely sensitive to light stimulation. They do not possess melanin and even a relatively small light exposure causes irreversible damage to their retina. The albino rat has been a model system for retinal damage testing, starting from the classical paper by Noell (Noell 1966). A fairly recent review article neatly summarizes these studies (De Vera Mudry 2013). In the light of these findings it is impossible to determine whether the observed effect is due to the stress response, or to the acute photodamage of retinal cells. The authors provide only a two sentence comment to explain this possibility (lines 299-301). Also, they observe

an adaptation effect upon consecutive sessions and explain it as adjustment to stress. An obvious alternative explanation is the decreased sensitivity of retina to the stimulus due to photodamage.
The electrophysiological data deserve publishing, but the design of the experiments does not provide satisfactory explanation of the observed effect. It is absolutely necessary to perform experiments that would distinguish between systemic stress response and the acute retina photodamage.

We thank the reviewer for the words and important comments regarding the photosensitivity of albino rats. We realize we should have provided a far more detailed description of our photic stress procedure and put it in better context as also mentioned by reviewer 1. We have now extended not only the Methods section but also extended the discussion on that point. Importantly our previous light exposure definition was not accurate enough and may have been misleading. We stated 120cd but should have provided either distance or lux in addition. Light intensity as candela was measured on the bucket floor approx. 60 cm. in distance away from the light source resulting in approx. 330 lux. Our lux regime is thus in accordance with procedures employed for anxiolytic tests with similar exposure times of albino models (Sakae et al. 2015, Mecerra Garcia et al. 2005, Sansri et al. 2014). It is in fact, on the lower level compared to experiments by Albani et al. 2015 (EPM, 400/1200 lux), Van der Schuren et al. 1995, (social play 572 lux), Rex et al. 1999, (open-field 1000 lux). Our lux regime is even below maximum constant light levels for some laboratory guides (<350 lux, The Laboratory Rat by Sharp & La Regina). Additionally, earlier studies have shown that in anaesthetized animals undergoing the same or similar stress procedures the CA1-Subiculum pathway experiences a long-lasting inhibition of LTP and facilitation of LTD (Commins & O'Mara 2000, Xu, Anwyl, Rowan 1997).

The absence of retinal tests in any or many other publications applying similar tasks and light intensities cannot per se be seen as a proof that there are no impairments. However, by observing the parameters in publications which investigate retinal damage in albino rats we observe that they have been performed under far more extreme light conditions over far longer periods. E.g. 5000 lux for 6h in Tanito et al. 2008 or 2700 lux for 1 day in Kaldi et al. 2003 or 1200-1400 lux for 8 hours in Organisciaket al. 2000, or 1700 lux for 1 day Li et al. 2003, Invest Opthalmil Vis Sci). Hence, in conclusion, we have not found any evidence in the literature that such short exposures as ours (even if repeated) with ~330 lux would have damaging effects to the eyes. We only found reports similar to Schremser et al. 1995 which reports rhodopsin changes in animals being raised in very low light intensities and then changed permanently to higher lux (~200) environments.

The very basic control would be the histological comparison between the retina of control animals (a parallel cohort never subjected to photic stress) and the experimental rats.
The authors could use different stressors (for example auditory) and compare their effects with the photic stress.
Another type of experiment would be to record HD activity in light vs dark conditions in order to identify visually driven HD cells, and then check whether this population is specifically affected by photic stress. If this was the case, the data would nicely support the concept that two populations of HD neurons may exist in parasubiculum, one responsible for the idiothetic input, and the other processing the allothetic stimuli provided by the visual system.

In light of our light stress we did and do not anticipate retinal damage and have no convincing evidence that we would have to expect one (see our comments above) as such additional control experiments would not be ethically justified. We acknowledge though the possibility as stated in the original and current extended discussion, that despite the evidence we cannot exclude retinal damage. The proposed experiment regarding the recording of HD activity in light vs. dark conditions would unfortunately not fully answer the questions either. Based on several lines of evidence it appears likely that the far lower head directional firing rates of neurons in the postsubiculum (compared to its HD input structures of the thalamus) present additional information modalities. A darkened state would also remove local cues (borders, walls) and peripheral cues (within the room) which may not be represented by pure visual inputs alone. We acknowledge and state in the manuscript that our conclusion would be stronger if one additionally tests alternative stressors and or stress protocols of varying lengths and strengths; however, we believe our novel data is presented in a precise and understandable manner and provides valuable information to the field.

We hope that our detailed responses are satisfying and provide more details on the procedure and methods applied. We apologize for inconsistencies and missing information in the original text.

2nd Editorial Decision 23 February 2018

Dear Dr. Passecker,

Your resubmitted manuscript was reviewed by one of our original external reviewers as well as by the Section Editor, Dr. Marco Capogna, and ourselves. We are pleased to inform you that will be accepted for publication in EJN after a couple of minor revisions.

Please ensure that your reporting of statistical data adheres to EJN guidelines, in particular we request precise values of P rather than inequalities (see EJN 2008, 28, 2363-2364;http://onlinelibrary.wiley.com/doi/10.1111/j.1460-9568.2008.06581.x/pdf). I would suggest also to remove 'its' from the title, it reads much better without it.

If you are able to respond fully to the points raised, we would be pleased to receive a revision of your paper within 30 days.

Thank you for submitting your work to EJN.

Kind regards,

John Foxe & Paul Bolam
co-Editors in Chief, EJN

Reviews:

Reviewer: 2 (Rafal Czajkowski, Nencki Institute of Experimental Biology, Poland)

Comments to the Author
The explanations are satisfactory. Two new issues have been noticed upon second review.
There is discrepancy between two descriptions of the photic stress paradigm. The authors state that control animals are kept in the home cage between recording sessions (lines 167, 168), but then when describing the timeline (lines 170, 171) they mention a black bucket.

There are problems with the axis labels in Fig 2 and 4.
In Fig. 2, the X axis should be named “Control 1 1st half” and the Y axis “Ctrl 1 2nd half” or something along these lines. Otherwise there is confusion between Fig 2 and Fig 3.
In Fig 4 the X and Y axis should be swapped (control session as X axis).

Authors’ Response 25 February 2018

Detailed response to the reviewers in blue:

Reviewer: 2
The explanations are satisfactory. Two new issues have been noticed upon second review.
There is discrepancy between two descriptions of the photic stress paradigm. The authors state that control animals are kept in the home cage between recording sessions (lines 167, 168), but then when describing the timeline (lines 170, 171) they mention a black bucket.

We thank the reviewer for spotting this discrepancy in the manuscript following the first revision and apologize for this mistake; as mentioned in the original version of the manuscript animals were kept in the home cage following the stress exposure and in case of two consecutive control recordings, animals stayed also in the home cage in between the control recordings. The latter was done to avoid unintentionally stressing the animals in a new environment (if not yet exposed to the black bucket) or have confounds that the animals associate the bucket with bright light if they have been exposed to photic stress already. For stress recordings we did not want to keep the animals in the black bucket after the light was turned off, as

the environment and smell association could potentially elongate the stress exposure (text was accordingly adapted in lines 157,170,174).

There are problems with the axis labels in Fig 2 and 4.
In Fig. 2, the X axis should be named “Control 1 1st half” and the Y axis “Ctrl 1 2nd half” or something along these lines. Otherwise there is confusion between Fig 2 and Fig 3.
In Fig 4 the X and Y axis should be swapped (control session as X axis).

We thank the reviewer for the helpful comments to improve the understanding of the figures and follow the instructions and adapted the Figures accordingly.
